# Association of *FTO* rs9939609 polymorphism with serum leptin, insulin, adiponectin, and lipid profile in overweight adults

**DOI:** 10.1080/21623945.2020.1722550

**Published:** 2020-01-29

**Authors:** Mahsa Mehrdad, Saeid Doaei, Maryam Gholamalizadeh, Majid Fardaei, Mohammad Fararouei, Mohammad Hassan Eftekhari

**Affiliations:** a Department of Clinical Nutrition, School of Nutrition & Food Sciences, Shiraz University of Medical Sciences, Shiraz, Iran; b Student Research Committee, Cancer Research Center, Shahid Beheshti University of Medical Sciences, Tehran, Iran; c Research Center of Health and Environment, Guilan University of Medical Sciences, Rasht, Iran; d Department of Medical Genetics, School of Medicine, Shiraz University of Medical Sciences, Shiraz, Iran; e HIV/AIDs Research Center, Shiraz University of Medical Sciences, Shiraz, Iran

**Keywords:** *FTO* gene, polymorphism, rs9939609, leptin, HDL

## Abstract

*FTO* gene polymorphisms are associated with obesity and food intake. This study aimed to investigate the association of *FTO* rs9939609 polymorphism genotypes with serum glucose, lipid profile and serum hormones level. This cross-sectional study was carried out on 196 randomly selected overweight adults. Anthropometric measurements including weight, height, body mass index (BMI), fat mass, and fat-free mass were assessed. Serum TGs, total cholesterol, HDL cholesterol, LDL cholesterol, glucose and insulin levels were measured. The *FTO* gene was Genotyped for rs9939609 polymorphism. Dietary intake was assessed by avalid 168-item semi-quantitative food frequency questionnaire (FFQ). The homozygotes for the *FTO* rs9939609 risk allele (A) had higher serum leptin (p = 0.005, F: 5.131) and lower HDL (p = 0.001, F: 7.687) level than TT genotype. The differences between TT and AT genotypes were not significant. The association remained significant for HDL level after adjustments for age and sex, calorie intake, physical activity, and BMI. The association between rs9939609 polymorphism genotypes and leptin was disappeared after adjustments for calorie intake and physical activity. In conclusion, rs9939609 risk allele was associated with higher serum leptin and lower HDL levels in overweight people. Further studies are warranted.

## Introduction

Obesity has been dramatically increased over the last two decades in both low and high-income countries [1]. Obesity is a risk factor for the other diseases such as cardiovascular disease, type 2 diabetes, hypertension, and psychological disorders [2–4]. The prevalence of obesity was also recently increased in Iran and more than 50% of the Iranian population is obese or overweight. Some genes such as the fat mass and obesity-associated (*FTO*) gene are strongly associated with obesity and overweight [4].

The *FTO* gene is expressed in many tissues, although the highest expression level of this gene is in the brain and hypothalamus. It is associated with the inflammatory state, food intake regulation and body metabolic rate [5–7]. Subsequently, many studies demonstrated that Single Nucleotide Polymorphisms (SNPs) such as rs9930609 are related to body mass index (BMI), obesity and related complications [8]. The PREDIMED study reported that the carriers of the A allele had the highest baseline body weight compared with TT genotype [9]. About half of the world’s population is the carriers of the risk allele [10]. More recent studies indicated that *FTO* gene variants were associated with food intake, satiety regulation and plasma level of leptin, adiponectin, and ghrelin hormones [11].

On the other hand, some biochemical factors such as serum hormones and glucose and lipid profile can act as a modulator of body weight and body composition. Leptin is a circulating hormone predominantly made and secreted into the circulation by adipose cells. Leptin regulates energy balance and food intake through difference pathways in both central and peripheral nervous systems [12]. Some studies indicated the possible association between *the FTO* gene and leptin and reported that *FTO* rs9939609 polymorphism is associated with leptin gene expression [12,13]. Moreover, evidence from several studies indicated the relationship between the *FTO* rs9939609 polymorphism with levels of total cholesterol (TC), LDL-C (low-density lipoprotein), triglyceride (TG), and HDL (high-density lipoprotein) [12–15]. Some studies reported that the presence of risk allele in *FTO* gene leads to a decrease in HDL level [15,16]. Therefore, we aimed to investigate the associations of rs9939609 polymorphism genotypes with leptin, adiponectin, insulin, serum glucose, and lipid profile in individuals with overweight.

## Material and methods

### Participants

This cross-sectional study was carried out from September 2016 to October 2017 on 196 randomly selected adults (50 men and 146 women) referred to the Shohadaye Valfajr Health Centre, Shiraz, Iran. The Inclusion criteria were defined as BMI between 24.9 and 29.9 kg/m^2^, age from 20 to 45 years, not participating in any weight management programmes during past two months and no weight loss greater than 5%. We excluded participants with alcohol and drugs consumption, smoking, certain weight-related diseases (including specific psychological or neurological disorders, thyroid disease, liver disease, renal failure, infectious and other specific diseases), and pregnant or lactating women. All subjects signed the consent form before participation in the study.

### Anthropometric measurements

Height was measured with a calibrated tape line fastened to a wall and without shoes with a precision of 0.5 cm. A bioelectric impedance analysis scale (BIA) (Tanita, Japan/BC-418) was used to measure body weight, Body Mass Index (BMI), body fat (BF), body fat percentage (BF%), skeletal muscle (SM), and skeletal muscle percentage (SM%) after entering their height, age and gender.

### Laboratory measurement

Serum TG, TChol, HDL, LDL, glucose and insulin levels were measured after 12 h of an overnight fasting. Serum level of leptin and adiponectin were measured using EDTA-anticoagulated tubes. Insulin, leptin and adiponectin level was determined by ELISA test using the specialized kit (LDN, Germany).

### Genotyping

DNA was extracted from whole peripheral blood sample using the DNA extraction kit (SinaPure DNA Kit, PR881612/EX6001/CinnaGen/Iran). DNA samples were stored at −20°C before genotyping. After DNA extraction, the concentration of the extract material was obtained by spectrophotometer NanoDrop (ND1000, USA). Genotypes for the *FTO* rs9939609 polymorphism (TT/AT/AA) were determined via amplification refractory mutation system polymerase chain reaction (ARMS-PCR).

### Dietary intake and physical activity

Usual dietary intakes of participants were assessed by a validated 168-item semi-quantitative food frequency questionnaire (FFQ) [17]. Face-to-face interviews were administered by a trained dietitian. All reported consumption frequencies were converted to grams per day by using household measures. The International Physical Activity Questionnaire (IPAQ) was used for measuring physical activity of participants through the face-to-face interview [18]. All results of the IPAQ were expressed and analysed as metabolic equivalents per minute (MET-minutes per week).

### Statistical analysis

ANOVA was used to describe demographic, anthropometric and hormone levels between three *FTO* genotype groups. The one-way multivariate analysis of variance (one-way MANOVA) was used to investigate the effect of *FTO* genotypes on serum insulin, leptin, and adiponectin levels and plasma FBS, LDL, HDL, TChol and TG level. The one-way multivariate analysis of covariance (MANCOVA) was used to adjust the effect of covariate variables. Univariate statistical tests were used to evaluate the differences between genotypic groups. We made a Bonferroni correction to account for multiple comparisons and p-value <0.006 were considered statistically significant. Data were analysed with SPSS software version 21.

### Ethics approval and consent to participate

This study has been approved by Local ethics review boards at Shiraz University of medical sciences (ir.sums.rec.1395.100).

## Results

Minor allele frequency (MAF) in this population was about 44.7%. Regarding *FTO* rs9939609 genotype, about half of the subjects were AT (n = 98), 30% of them were TT (n = 60) and about 20% of them were homozygote for the known risk allele of obesity (n = 38). FM was significantly different in three genotype groups (p = 0.001). Genotypes AA and AT had higher calorie intake and lower physical activity compared to TT. However, the differences between genotypes were not statistically significant. Details of subjects’ characteristics are presented in Table 1.

**Table 1.** Participants’ characteristics by FTO rs9939609 genotypes (N = 196)

Variables	TT (n = 60)	AT (n = 98)	AA (n = 38)	P
Male sex (%)	15(25)	25(25.51)	10(26.3)	0.989
Age(years)	33.43(±6.461)	32.99(±6.488)	34.08(±5.961)	0.664
Weight(kg)	72.140(±9.8058)	72.618(±9.1667)	75.262(±9.3294)	0.241
Height(m)	163.983(±9.8402)	163.980(±9.4112)	165.816(±9.0609)	0.564
BMI(kg/m2)	26.7086(±1.10977)	26.9072(±1.03883)	27.2864(±1.33132)	0.047
Fat Mass (kg)	21.380(±3.9137)	22.160(±3.3318)	24.363(±4.2223)	0.001*
FM%	30.0847(±6.07727)	31.0358(±5.94178)	32.7175(±6.23811)	0.112
FFM (kg)	50.7600(±10.22795)	50.4586(±10.25310)	50.8989(±9.67006)	0.968
FFM%	69.9153(±6.07727)	68.9642(±5.94178)	67.2825(±6.23811)	0.112
Calorie intake	1966.68(±357.334)	2027.72(±368.478)	2139.00(±396.456)	0.083
FBS(mg/dl)	86.95(±8.490)	89.18(±9.738)	91.42(±11.568)	0.084
LDL-C(mg/dl)	96.90(±20.755)	102.75(±16.100)	103.82(±18.475)	0.088
HDL-C (mg/dl)	47.20(±9.950)	42.82(±8.175)	40.87(±6.751)	0.088
TChol(mg/dl)	183.13(±29.514)	192.42(±23.446)	199.50(±25.438)	0.008
TG(mg/dl)	113.87(±48.315)	118.03(±27.235)	118.74(±29.388)	0.724
Insulin(μIU/dl)	7.93(±2.821)	8.41(±2.638)	8.55(±2.177)	0.425
Leptin(ng/dl)	44.6282(±23.35567)	50.1287(±23.09528)	59.3313(±16.97927)	0.007
Adiponectin(ng/dl)	11.6505(±5.05365)	11.2051(±15.26091)	7.5697(±8.19622)	0.198

# Abbreviations: BMI, body mass index; HDL, high-density lipoprotein; FFM, fat-free mass; LDL, low-density lipoprotein; TC, total cholesterol; TG, triglycerides; FBS, fasting blood sugar.

*P-value 0.002.

Association of *FTO* rs9939609 polymorphism genotype with the level of hormones, FBS, and lipid profile is presented in Table 2. There was a significant difference between *FTO* genotype groups for serum leptin and HDL levels. This relationship remained significant after adjustment for age and sex (p = 0.007 and p = 0.001, respectively). This association disappeared for leptin after further controlling for calorie intake and physical activity (p = 0.030) but remained significant for HDL-c (p = 0.000). The results did not substantially change after further adjustment for BMI.

**Table 2.** Association of FTO genotypes (TT, AT, & AA) with the level of hormones, FBS, and lipid profile using multivariate analysis (n = 196)

	Model 1		Model 2		Model 3		Model 4	
variables	F	P	F	P	F	P	F	P
Insulin(μIU/dl)	0.858	0.425	0.846	0.431	0.952	0.388	0.901	0.408
Leptin(ng/dl)	5.131	0.005	5.185	0.005	3.380	0.036	2.901	0.057
Adiponectin(ng/dl)	1.632	0.198	1.447	0.238	1.377	0.255	1.430	0.242
FBS(mg/dl)	2.503	0.084	2.447	0.089	2.338	0.099	1.885	0.155
LDL(mg/dl)	2.456	0.088	2.642	0.074	3.011	0.052	2.974	0.053
HDL(mg/dl)	7.687	0.001	7.849	0.001	8.140	0.000	8.110	0.000
TChol(mg/dl)	4.999	0.008	4.932	0.008	3.560	0.030	3.793	0.024
TG(mg/dl)	0.323	0.724	0.329	0.720	0.864	0.423	0.750	0.474

^#^Abbreviations: BMI, body mass index; HDL, high-density lipoprotein; FFM, fat-free mass; LDL, low-density lipoprotein; TC, total cholesterol; TG, triglycerides; FBS, fasting blood sugar.

Model 1: Adjusted for age& sex; Model 2: Additional adjustments for calorie & physical activity; Model 3: Further adjustment for BMI.

*P-value 0.006.

Tukey tests for recognize differences between three genotypes identified a significant difference between clinical parameters and genotypes of *FTO*. Subjects with AA genotype for the rs9939609 polymorphism had significantly different serum HDL-c and leptin levels than those with TT genotype. Individuals with AA genotype had significantly higher serum leptin and lower serum HDL-c levels compared to those with TT genotype. The difference between carriers TT and AT was significant only for HDL-c, not for Leptin (Table 3).

**Table 3.** Tukey test for comparison the Clinical Parameters between three genotypes (TT, AT, & AA)

Variables	TT (n = 60)	AT (n = 98)	P value	AA (n = 38)	P value
Insulin(μIU/dl)	1	−0.48	0.505	−0.62	0.488
Leptin (ng/dl)	1	−5.5005	0.285	−14.7031*	0.005
Adiponectin (ng/dl)	1	.4454	0.971	4.0807	0.218
FBS(mg/dl)	1	−2.23	0.344	−4.47	0.072
LDL(mg/dl)	1	−5.85	0.121	−6.92	0.158
HDL(mg/dl)	1	4.38	0.005	6.33*	0.001
TChol (mg/dl)	1	−9.29	0.074	−16.37*	0.007
TG(mg/dl)	1	−4.16	0.754	−4.87	0.784

# Abbreviations: HDL, high-density lipoprotein; LDL, low-density lipoprotein; TChol, total cholesterol; TG, triglycerides; FBS, fasting blood sugar.

*P-value 0.006.

The univariate tests were used to adjust the effect of confounding variables on the significant relationships between three genotypes of *FTO* and leptin and HDL-c levels. The results of the univariate tests to adjust the effect of confounding variables on the significant relationships between three genotypes of *FTO* with leptin and HDL-c levels reported that adjustment for age and sex (model 1), calorie intake and physical activity (model 2), and BMI (model 4) did not change the results. The significant association between *FTO* and leptin was disappeared after controlling for calorie intake and physical activity (Table 4).

**Table 4.** Univariate tests for comparison the level of Leptin and HDL between three genotypes

	Model 1			Model 2			Model 3		
Variables	TT	AT	AA	TT	AT	AA	TT	AT	AA
Leptin (ng/dl)	1	−5.532	−14.760*	1	−4.734	−12.371	1	−4.416	−11.552
P Value		0.128	0.002		0.193	0.01		0.225	0.017
HDL (ng/dl)	1	4.362*	6.293*	1	4.565*	6.636*	1	4.591*	6.703*
P Value		0.002	0		0.001	0		0.001	0

^#^Abbreviations: HDL, high-density lipoprotein.

Model 1: Adjusted for age& sex; Model 2: Additional adjustments for calorie & physical activity; Model 3: Further adjustment for BMI.

*P-value 0.006.

## Discussion

The findings identified that the *FTO* rs9939609 risk allele was associated with higher leptin and lower HDL-c levels. This association remained significant for HDL-c level but disappeared for leptin level after adjustments for calorie intake and physical activity. The MAF in our population was approximately close to MAF reported in Europeans as Caucasian ethnicity (45%) [19,20]. It was reported that the overall estimation of MAF in Caucasians is about 44% [21].

The lowest and highest MAF reported in various populations are, respectively, observed in East Asians (~12%) [22] and West Africa (~52%) [19]. It should be noted that the frequency of AA genotype was reported variously in different ethnicities, from lowest frequencies as in aboriginals (5%) [20] to highest in Pakistan (~30%).

Since MAF in our population is close to the highest reported MAF in the world, this polymorphism might considerably affect obesity and the related complications in this region. In another study in Iran, MAF for healthy individuals was 42%, and the frequency of AA genotype was 16% [23], which these findings were approximately in line with the results of the present study. In accordance with our findings, some studies found that *FTO* rs9939609 polymorphism is strongly associated with insulin sensitivity and plasma leptin level [24,25]. Another study conducted on the association between *FTO* gene rs9939609 polymorphism and obesity-related hormones reported that the carriers of the A-allele had higher leptin levels, but this relationship disappeared after adjustment for BMI [26,27]. In our study, there was no association between leptin level and *FTO* polymorphism after adjustment for calorie intake and physical activity. By contrast, Duicu et al. found no association between *FTO* rs9939609 and leptin level [28]. However, their participants were children. It is possible that the association between *FTO* genotype and serum leptin level changes through the lifespan. In line with the previous studies [26,27], the association between *FTO* genotype and leptin was disappeared after adjustments for BMI-related factors. It is plausible that FTO risk allele can increase the level of serum leptin via increasing the BF and BMI. FTO rs9939609 polymorphism is reported to be associated with REE [29]. The leptin also plays an important role in the regulation of resting energy expenditure (REE). We hypothesized that the effects of FTO on REE can be mediated by leptin. As mentioned before, some studies indicated the possible association between *the FTO* gene and leptin and reported that *FTO* rs9939609 polymorphism is associated with leptin gene expression [12,13].

Moreover, the results indicated that the *FTO* rs9939609 polymorphism was associated with HDL-c levels. The carriers of the A-allele of rs9939609 polymorphism had lower HDL-c level than carriers of T-allele. In accordance with our findings, Zhang et al. reported that carriers of the A-allele of rs9939609 had lower HDL-c compared with controls [30]. Another study reported that homozygotes for the A-allele of *FTO* rs9939609 polymorphism had 1.25-fold lower HDL-c level compare with TT genotype [16].

Lappalainen et al. also found that the individuals carrying A-allele of rs9939609 especially those with AA genotype showed lower HDL-c level (p = 0.007) in comparison to those with TT genotype [31]. The A-allele of *FTO* rs9939609 polymorphism in individuals with diabetes was associated with lower HDL-c (p = 0.008) and higher TG level (p = 0.007), and also the risk of cardiovascular disease was increased in the carriers of A-allele [32]. In contrast with our results, some studies found no significant association between *FTO* polymorphism and HDL-c level [33]. However, both of these studies were done on children. It is possible that the association between *FTO* genotype and serum HDL-c level is also variable through different ages.

The present study reported that the association between various genotypes of *FTO* rs9939609 polymorphism and HDL-c remained significant after adjustment for calorie intake, physical activity and BMI. These results may indicate a strong association between HDL-c and *FTO* genotype. However, the exact pathway by which *FTO* polymorphism is associated with HDL-c level is not specified yet and requires further investigations. Finally, we can claim that the effect size of A-allele on HDL-c and leptin is approximately close to the effect size in the European population; thus, we can conclude that the associations and effect size of this variant in this population is similar to those in Europeans, since our population is Caucasians as Europeans [16,27]. Evidences showed that this gene can regulate lipid profile through hepatic signalling pathways; however, the exact mechanism is not yet understood [34,35]

Since HDL-c level is strongly associated with non-communicating diseases, based on these findings, carriers of A-allele might be more susceptible to metabolic disturbances and subsequently affected by non-communicating diseases such as metabolic syndrome, cardiovascular diseases and other chronic diseases in comparison to non-carriers. In addition, the associations of this variant are different across various regions, ethnicities, and age groups. Therefore, it is strongly recommended to replicate this type of studies across different populations to better understand the associations and effect sizes. Although one of the limitations of this study were the lack of a group with normal BMI to determine the exact association of this variant with obesity and its related complications, our primary aim was to compare the effect of this variant between different genotypes of this polymorphism. We matched the participants based on various variables that may confound the difference of effect size for the alleles, such as BMI, calorie intake, ethnicity and physical activity. However, further studies are needed through different BMI ranges to clarify the exact effect, and also related mechanisms, of this variant on the above indices. One of the strengths of this study was considering various confounders in statistical analysis to obtain more accurate and pure results.

## Conclusion

This study found that the homozygotes for the rs9939609 risk allele (A) had significantly higher serum leptin and lower HDL-c level than those with TT genotype. Although, this result remained significant only for HDL-c level after adjustment for BMI. We can conclude that AA genotype might be more susceptible to non-communicable diseases in comparison to those with TT genotype. Further studies are needed to increase our understanding of the association for *FTO* rs9939609 polymorphism with lipid profile and leptin.
